# The Existence of a Hypnotic State Revealed by Eye Movements

**DOI:** 10.1371/journal.pone.0026374

**Published:** 2011-10-24

**Authors:** Sakari Kallio, Jukka Hyönä, Antti Revonsuo, Pilleriin Sikka, Lauri Nummenmaa

**Affiliations:** 1 School of Humanities and Informatics, University of Skövde, Skövde, Sweden; 2 Centre for Cognitive Neuroscience, University of Turku, Turku, Finland; 3 Department of Psychology, University of Turku, Turku, Finland; 4 Brain Research Unit, Low Temperature Laboratory, Aalto University School of Science, Espoo, Finland; 5 Department of Biomedical Engineering and Computational Science, Aalto University School of Science, Espoo, Finland; 6 Turku PET Centre, University of Turku, Turku, Finland; Kyushu University, Japan

## Abstract

Hypnosis has had a long and controversial history in psychology, psychiatry and neurology, but the basic nature of hypnotic phenomena still remains unclear. Different theoretical approaches disagree as to whether or not hypnosis may involve an altered mental state. So far, a hypnotic state has never been convincingly demonstrated, if the criteria for the state are that it involves some objectively measurable and replicable behavioural or physiological phenomena that cannot be faked or simulated by non-hypnotized control subjects. We present a detailed case study of a highly hypnotizable subject who reliably shows a range of changes in both automatic and volitional eye movements when given a hypnotic induction. These changes correspond well with the phenomenon referred to as the “trance stare” in the hypnosis literature. Our results show that this ‘trance stare’ is associated with large and objective changes in the optokinetic reflex, the pupillary reflex and programming a saccade to a single target. Control subjects could not imitate these changes voluntarily. For the majority of people, hypnotic induction brings about states resembling normal focused attention or mental imagery. Our data nevertheless highlight that in some cases hypnosis may involve a special state, which qualitatively differs from the normal state of consciousness.

## Introduction

Does a hypnotized person enter a special hypnotic state that is completely outside the range of normal mental states and cognition? This question has been under debate throughout the history of hypnosis research [1], [2], [3]. Major psychological models of hypnosis called the Non-State View theories explicitly reject the existence of a special hypnotic state [2], [3]. Instead, they assume that all hypnotic phenomena involve only cognitive and neural states similar to those occurring outside of hypnosis [3]. The opposing theoretical view called the State-View posits that a hypnotic state exists which differs qualitatively from the normal waking baseline state [2], [3]. So far, no hypnotic state fulfilling objective empirical criteria has ever been convincingly demonstrated. Consequently, a special altered hypnotic state is currently considered by some researchers to be merely a popular myth in psychology [4]. Nevertheless, it is generally agreed that the existence of a hypnotic state could be defined empirically by behavioural criteria reflecting changed information processing that cannot be imitated or simulated by nonhypnotized control subjects [2], [3].

Functional brain imaging studies of the state following hypnotic induction without any further suggestions (pure hypnosis) have not been able to resolve the questions concerning the existence of the hypnotic state or its neural basis [5]. The results from different studies have diverged somewhat. In part, this may be due to the lack of a common definition for the presence of hypnosis. Furthermore, in most of the experiments the subjects were engaged in different tasks during data acquisition, which hampers the possibility of infering the effects of the hypnotic state as such. For example, in a study utilising positron emission tomography (PET) it was found that a standard hypnotic induction resulted in an activation pattern that included the brainstem, the thalamus, the anterior cingulate cortex, the right inferior frontal gyrus and the right inferior parietal lobule [6]. In another PET study [7] hypnosis was related to the activation of a set of cortical areas involving occipital, parietal, precentral, premotor, and ventrolateral prefrontal and anterior cingulate cortices. However, in the former study the subjects' left hand was immersed in warm or painfully hot water during the experiment, whereas in the latter the subjects were asked to recall pleasant autobiographical memories during hypnosis. A study employing event-related fMRI and EEG-coherence analysis found evidence of decoupling between the anterior cingulate cortex and the left dorsolateral prefrontal cortex [8] during Stroop task performance. On the other hand, a recent experiment [9] studying the “default mode” network (areas active in the absence of goal directed activity) demonstrated that during hypnosis and at rest (i.e., while not performing any specific tasks) high suggestible subjects had decreased activation in a number of prefrontal regions, including the ACC and dorsolateral prefrontal cortices.

Despite these methodological differences in different studies converging evidence of the involvement of the anterior cingulate cortex (ACC) and dorsolateral prefrontal cortical areas (dlPFC) has emerged [5], [10]. These areas play a crucial role in the initiation and motivation of goal-directed behaviours [11] as well as in attention-related functions [12]. In addition, the ACC has known anatomical connections with the frontal eye field (FEF) and supplementary eye field (SEF) [13] and thus plays a prominent role in regulating eye-movements [14]–[17]. Similarly, the dlPFC, through its connections with the superior colliculi and FEF, is involved in the control of eye movements [14].

Thus, the proposed functions of the frontal areas and especially the ACC match well with the changes in behaviour that the hypothetical hypnotic state is assumed to bring about e.g. intensification of focused attention and attenuation of the planning functions as well as lack of spontaneity and initiative [18], [19].

In the present study we focused on a classical behavioural marker of hypnosis, the “trance stare” (Hereafter *Hypnotically Induced Stare,* HIS, since we do not want to advocate the use of the term “*trance”*, since it is a vague concept lacking definition in scientific literature). It is a glazed look in the eyes accompanied by a highly reduced eye-blinking rate [20]–[22]. Weitzenhoffer [18] has described this phenomenon flowingly:

“…The subject seems to be staring, unblinking, possibly unseeing, at some indefinite point in space… If he is facing you, you may have the impression he is staring right through you at something further away. It is one of signs that have possibilities for research” p.182.

However, a conspicuous HIS is rare and little scientific research has been conducted on the oculomotor changes associated with hypnosis [23], [24]. Nevertheless, this phenomenon has by some researchers been previously considered to be one of the main behavioural signs of a hypnotic state [18] and it is widely used in the popular culture to represent the presence of hypnosis.

In this case study our aim was to test the state theory of hypnosis. We chose to focus on automatic forms of eye behavior because in a crucial way they are regulated by the frontal structures of the brain, such as the ACC and dlPFC [14], on which hypnosis has been shown to have an effect [5]. Furthermore, even though some previous preliminary experiments have shown promising results (see e.g. ref [24]), eye movements during hypnosis have not been studied with current high-resolution eye-tracking methodology. The view that hypnosis can involve an altered state would be supported if significant changes were found in automatic eye behavior between baseline and hypnosis.

## Methods

### Ethics statement

The research was conducted according to the ethical standards of the American Psychological Association (APA) and approved by the Ethics Committee of the University of Turku, Finland (statement 14/2011). All subjects gave their written informed consent for participation in the study. Participant TS-H has seen the manuscript, figures, and movie files and has provided a written consent for publication.

### Participants

#### TS-H

TS-H is a 43-year-old right-handed female office worker with normal vision. In a widely used scale measuring hypnotic susceptibility [19] TS-H scored the maximum of 12 points. She thus experiences vivid visual and acoustic hallucinations in response to suggestions during hypnosis. As TS-H has previously participated in several hypnosis experiments [25]–[28] there is already a body of research providing evidence that her brain function is considerably altered during hypnosis. More specifically, TS-H showed larger mismatch negativity (MMN) responses during hypnosis suggesting altered information processing in the brain at an early preattentive level [25]. In another study [26] hypnosis was found to lead to an altered functional synchronicity in the electrophysiological activity of the brain. In addition, hemispheric EEG asymmetry was observed during hypnosis with dominance of the right hemisphere, whereas no hemispheric dominance was observed in the baseline state [27].

**Figure 1:**
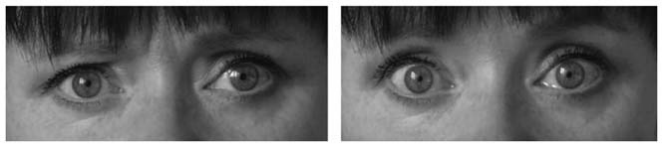
The Hypnotically Induced Stare (HIS). Subject TS-H in normal baseline state (a) and during hypnosis (b).

TS-H possesses further qualities that make her an ideal subject for experiments where hypnosis is being used. She responds immediately and invariably to a posthypnotic suggestion about entering hypnosis (for a further description of a posthypnotic suggestion and how it was used in this study, see Text S1) by spontaneously showing clear behavioural signs of a hypnotic state described by Weitzenhoffer [18]. She becomes immobile, her eye-blink rate decreases and her eyes appear to lack a fixation point (see Figure 1). TS-H has no history of any neurological or psychiatric illnesses and has a normal psychometric profile. See Table S1 and Text S2 for a more detailed description of the neuropsychological examination of TS-H.

#### Control group

An age-matched group of 14 volunteers (6 males, 8 females, mean age (± s.d.) of 42.6±5.6 years) with no history of neurological or psychiatric illness served as controls.

### Stimuli

**Figure 2:**
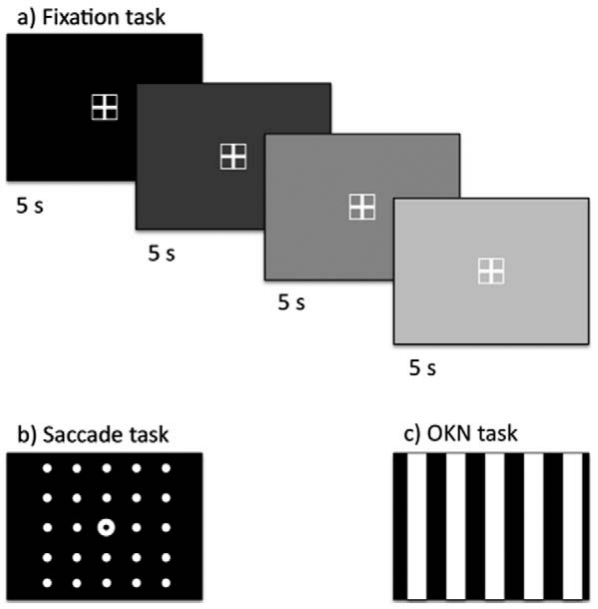
Summary of experimental tasks. This figure shows the three tasks and stimuli that were used in this experiment. In the Fixation task (a) the participants were instructed to maintain a stable fixation on a 2×2° target cross for 5 s at time. The luminosity of the background was manipulated (black, 30% white, 60% white or 90% white) across trials in order to induce changes in the pupil size. There were ten trials per luminosity condition presented in a random order. In the Saccade task (b) each trial begun with a fixation circle displayed at the centre of the screen for a random period between 100 and 500 ms. Next, the target circle appeared on the screen and the participant had to perform a saccade on the circle as fast and accurately as possible. Targets could appear at 24 different locations (as shown in b), at five different eccentricities. Twelve trials with targets at each eccentricity were presented in a random order. In the Partial field OKN task (c) the participants had to maintain stable fixation at the centre of the screen while a black and white grating moved towards left or right across the screen for 8.5 seconds on each trial. Two different speeds (4 or 6 degrees per second) were used. A total of 32 trials (six per condition) were presented in a random order. This task elicits the optokinetic reflex, an automatic to-and-fro oscillation of “slow pursuit and quick return” pattern of eye movement.

We presented a set of well-established oculomotor tasks (Figure 2) that trigger automatic eye behaviour i.e. the pupillary reflex, programming a saccade to a single target and the optokinetic reflex [14]–[17].

### Apparatus

Stimuli were presented on a 21″ monitor (120 Hz refresh rate) with a 3.2 GHz Pentium IV computer. Participants' eye movements were recorded with an EyeLink1000 eyetracker (SR Research, Mississauga, Ontario, Canada) connected to a 2.8 GHz Pentium IV computer. The sampling rate of the eyetracker was 1000 Hz, and the spatial accuracy was better than 0.5°, with a 0.01° resolution in the pupil-tracking mode. All subjects had their head supported by a chin and forehead rest in order to minimize head motion. In all tasks each trial began with a drift correction. A fixation circle appeared at the centre of the screen, and the participant had to focus his/her gaze at the centre of the circle. When the participant's eye was fixated on the circle, the experimenter initiated the trial. In all tasks, the actual experiment was preceded by practice trials.

### Analysis

For the Fixation task, mean fixation count, blink count, pupil size and fixation duration were measured and averaged for each background luminosity condition. For the Saccade task, only the first saccade on each trial was analyzed, and saccades with latencies less than 80 ms were considered as anticipations and omitted. Saccade latencies, amplitudes, durations and velocities were measured, and averaged for each eccentricity condition. For the optokinetic nystagmus (OKN) task, mean saccade amplitude, saccade velocity, fixation duration and OKN rate (saccades per second) were measured and averaged for each combination of grating scroll direction and speed.

### Procedure

All the subjects (including TS-H) were exposed to the eye movement tasks for the first time when they were tested and thus they were all naïve to the tasks. None of the subjects had training in any particular eye movement patterns and had not participated in any previous eyetracking experiments. Thus the present data arise from the very first eye movement experiment in which they had ever participated. TS-H participated in two separate testing sessions on two consecutive days. In both sessions, she performed all the tasks in two conditions, normal condition (NC) and hypnosis condition (HC). In HC, hypnosis was induced at the beginning of each task and cancelled when the task was completed. See Movie S1 for a video-clip where hypnosis is induced and cancelled for TS-H as done in this experiment. The only difference between the NC and HC was that in the former the hypnotist (author S.K.) said “*OK*” just before the task began whereas in the latter he uttered the cue word *“hypno”* inducing hypnosis. The same procedure of inducing and cancelling hypnosis has been used in previous studies with TS-H [25]–[28]. The order of these conditions was counterbalanced across sessions, and data were averaged across sessions. Control subjects were tested individually in a single session in which they performed all the tasks twice: in a normal condition (NC) and in hypnosis simulation condition (HSC). The control group received the task instructions in the NC exactly the same way as TS-H. In HSC, the control group was requested to attempt to simulate how a hypnotized person performs the tasks the best they could. In order to be able to mimic the appropriate behavioural outcome (staring eyes and almost petrified stance) the subjects were first shown a video clip of how TS-H behaves upon hearing the cue word (the posthypnotic suggestion). Then the control subjects were given a verbal description of how the eyes of TS-H move during the specific task and, in addition, shown a video clip of the eye movements of TS-H in the respective task during hypnosis. For the control group the same cue word “*hypno*” served as a sign to start simulating hypnosis. The order of the two conditions was counterbalanced across subjects.

## Results

**Figure 3:**
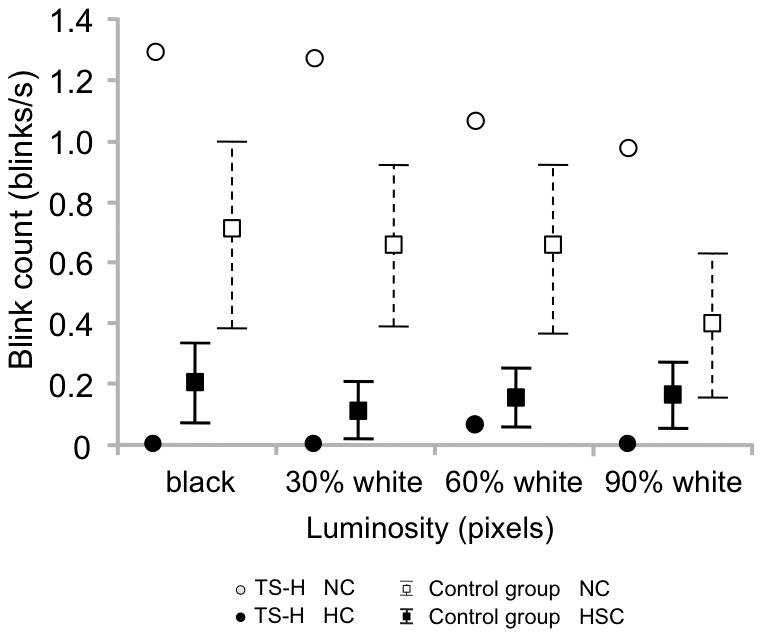
Blink count in the Fixation task. Means (TS-H, The control group) and the 95% Cls (The control group) for blink count in the Fixation task. The four background luminance manipulations in the Fixation task are marked as relative percentages from black to white background (black background-30% white background-60% white background-90% white background).

In the Fixation task, TS-H showed a markedly reduced eye-blinking rate (Figure 3) in HC (0.012 ± 0.04 blinks/s) as compared to NC (1.18 ± 0.63 blinks/s). Although some control subjects could mimic rather well this external feature of the “trance stare”, at the group level the changes were far less marked (0.58±0.56 blinks/s in NC and 0.15±0.23 blinks/s in HSC).

**Figure 4:**
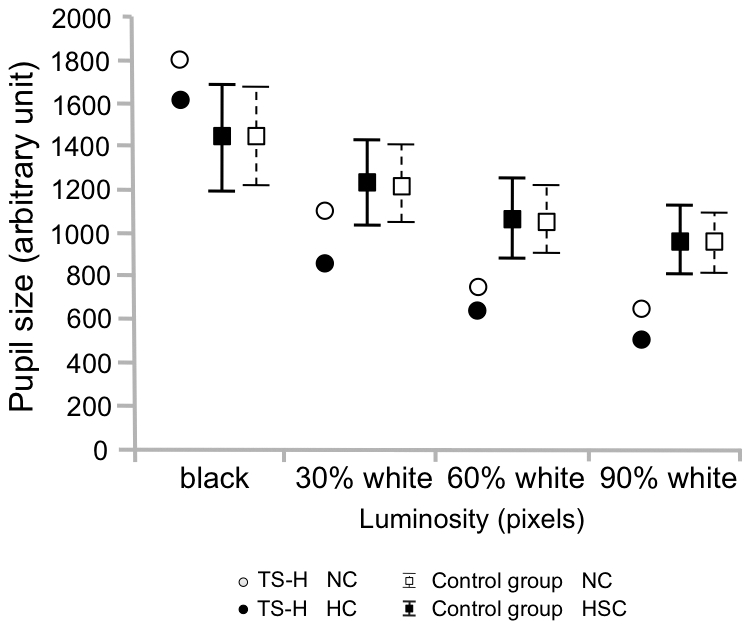
Pupil size in the Fixation task. Means (TS-H, The control group) and the 95% CIs (The control group) for pupil size in the Fixation task. The four background luminance manipulations in the Fixation task are marked as relative percentages from black to white background (black background-30% white background-60% white background-90% white background).

Contrary to the control group, the pupil size (Figure 4) of TS-H was slightly diminished during HC (913.1 ± 464.3 arbitrary unit) as compared to NC (1071.6 ± 572.7), whereas the reactions of the pupil size to changes in luminance were similar in TS-H and the control group. See also Table S2 for an overview of the changes between NC and HC for TS-H and the control group in all measured variables in the Fixation task.

**Figure 5:**
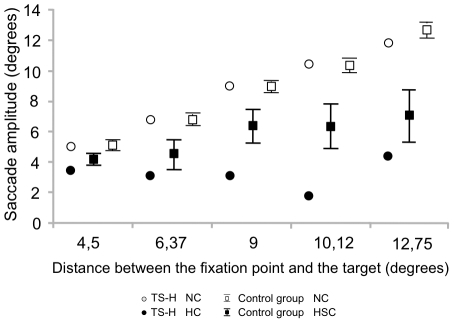
Saccade amplitude in the voluntary Saccade task. Means (TS-H, The control group) and the 95% Cls (The control group) for the mean saccade amplitude in the voluntary Saccade task. The saccade amplitudes are presented as a function of the 5 target eccentricities.

In the Saccade task hypnosis led to a special pattern of eye movements (Figure 5). In HC, TS-H performed only short saccades toward the target regardless of the distance from the fixation point. This “creeping” pattern of short saccades (Presented in Movie S2) was difficult to simulate by the control group since their fixation tended to automatically gravitate to the target (Presented in Movie S3). Mean saccade amplitude for TS-H was 3.3 ± 1.6 degrees/s in HC as compared to 8.6 ± 2.7 degrees/s in NC and for the controls the respective figures were 5.5 ± 2.1 and 8.8 ± 0.5 degrees/s. See also Table S3 for an overview of the changes between NC and HC for TS-H and the control group in all measured variables in the Saccade task.

**Figure 6:**
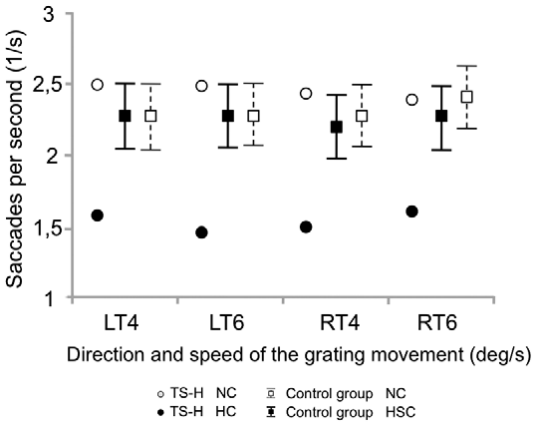
Number of saccades in the OKN task. Means (TS-H, The control group) and 95% CIs (The control group) for the number of saccades in the OKN task. In the OKN task the grating moved either towards left (LT) or right (RT) at two speeds (4 and 6 degrees/s).

**Figure 7:**
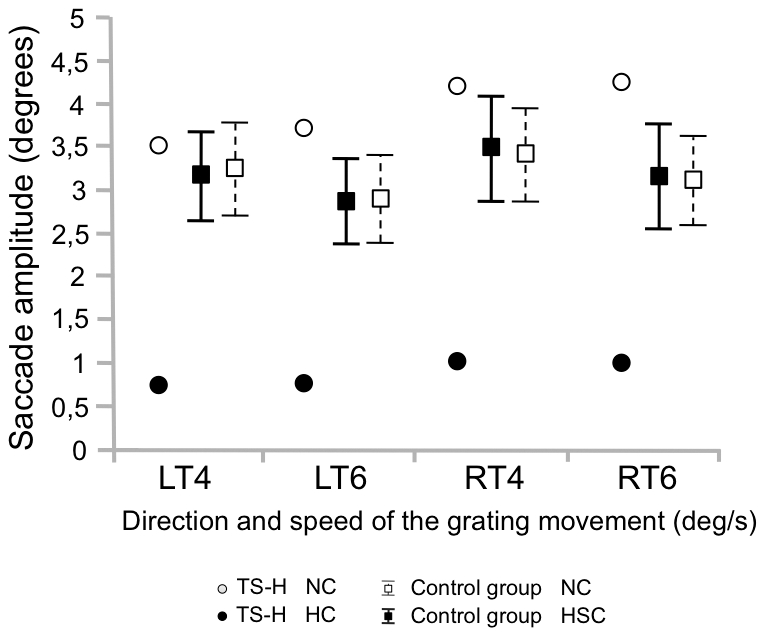
Saccade amplitude in the OKN task. Means (TS-H, The control group) and the 95% CIs (The control group) for saccade amplitude in the OKN task. In the OKN task the grating moved either towards left (LT) or right (RT) at two speeds (4 and 6 degrees/s).

The oculomotor changes during hypnosis in TS-H were largest in the OKN task, in which the number, size and speed of eye movements decreased and eye fixation duration increased dramatically. The number of saccades per second (see Figure 6) diminished in TS-H from 2.5±0.1 in NC to 1.5±0.1 in HC but remained unchanged for the control group (2.31±0.5 in NC and 2.27±0.4 in HSC). In TS-H the saccade mean amplitude (Figure 7) decreased from 3.9±0.4 degrees in NC to 0.89±0.16 degrees in HC, whereas the control group showed no change between NC and HC (3.16±0.3 and 3.16±0.3 degrees, respectively).

**Figure 8:**
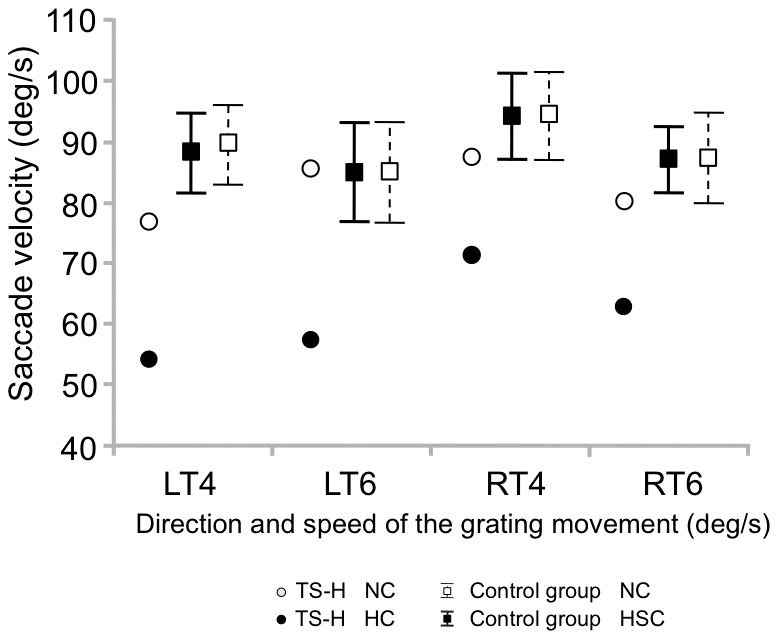
Eye saccade velocity in the OKN task. Means (TS-H, The control group) and the 95% CIs (The control group) for eye saccade velocity in the OKN task. In the OKN task the grating moved either towards left (LT) or right (RT) at two speeds (4 and 6 degrees/s).

**Figure 9:**
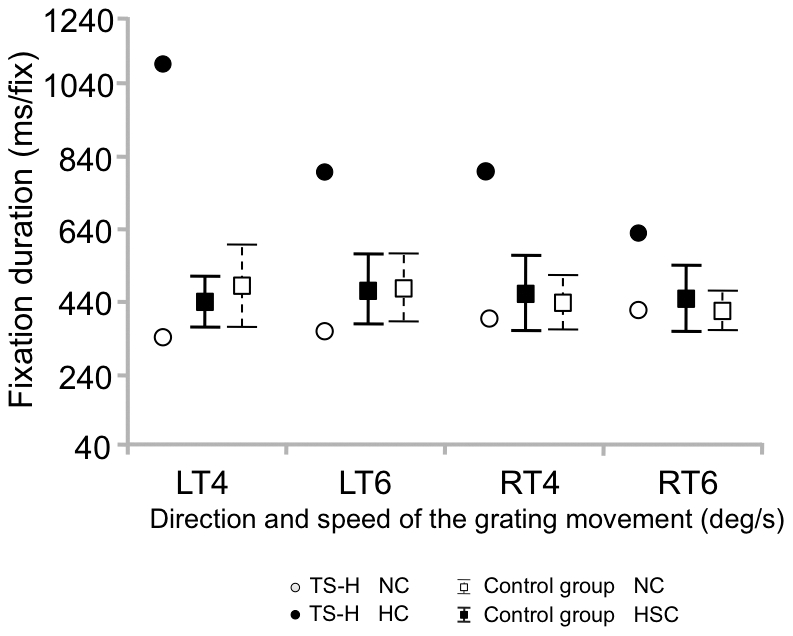
Eye fixation duration in the OKN task. Means (TS-H, The control group) and the 95% CIs (The control group) for eye fixation duration in the OKN task. In the OKN task the grating moved either towards left (LT) or right (RT) at two speeds (4 and 6 degrees/s).

The mean velocity of saccades (Figure 8) in TS-H decreased from 82.6±4.2 degrees per second in NC to 61.9±7 degrees per second in HC but did not change for the control group (88.9±3.8 degrees per second in NC and 88.7±3.5 degrees per second in HSC). The mean eye fixation duration (in ms) in this task (Figure 9) increased in TS-H from 361.3±40.8 in NC to 819.8±429 in HC (note the high variability during hypnosis indicating that shorter fixations also occur), while there was a large overlap in the control group between NC (432.6±25.6) and HC (439.1±14.1). The pattern of eye movement alterations in the OKN task concerning the number and size of saccades as well as fixation duration was far beyond the level that could be reached even by the best simulator (see Movie S4 and Movie S5 for TS-H's and a control subject's performance in the task and Table S4 for an overview of the changes between NC and HC for TS-H and the control group in all measured variables in the OKN task).

## Discussion

We showed that a Hypnotically Induced Stare (HIS) is accompanied by large, objective and inimitable changes in the patterns of eye movements in the case TS-H. The amplitude, velocity and frequency of reflexive saccades were radically suppressed, and the fixation time was increased. Also the pupil size of TS-H diminished during the hypnosis condition.

This study provides the first demonstration of the existence of a special hypnotic state, fulfilling all the strict empirical criteria for such a state (immediate induction and cancellation, objective confirmation through measurements, and inimitability). Support for hypnosis as a special state has so far escaped objective measurement and verification probably because it only occurs in a small proportion of the population. Also research focusing on eye-movements during hypnosis has not raised much interest so far.

Our results are in line with findings from both eye movement and hypnosis research. Eye movement studies have demonstrated that the maintenance of visual fixation and suppression of reflexive saccades involves activity in the ACC and dlPFC [29]–[30]. Hypnotic induction has been shown to involve changed patterns of activation in the same cortical areas [6]–[7], [9]. The putative role of these prefrontal areas in the hypnotic state is further elaborated by our previous results with TS-H. Two studies [26]–[27] utilizing EEG have revealed changes in the frontal areas of the brain. Fingelkurts et al. [27] found that in the prefrontal EEG channels the composition of brain oscillations included spectral patterns during hypnosis that were significantly different from those observed during non-hypnosis. Another study [26] demonstrated that with TS-H all frequency generated neuronal assemblies from the frontal areas (especially from the left hemisphere) lost active functional connections with the rest of the cortex during hypnosis. Thus, all the above seems to suggest that the hypnotic state is accompanied by changes in the prefrontal areas of the brain, especially in the ACC and dlPFC. The functional significance of these brain areas in the induction and/or maintenance of the hypnotic state remains to be elucidated in future studies employing brain imaging techniques.

The next task is to establish how prevalent the HIS phenomenon is and whether hypnosis is an entirely different psychological and neural phenomenon for those who enter the objectively confirmable hypnotic state, and for the vast majority of people who don't. The underlying assumption in modern hypnosis research has been that hypnosis and the hypnotic state represent a graded phenomenon that occurs as a normally distributed continuum across the population [31]. The current praxis of using behavioural scales [19], [32] when selecting subjects for hypnosis experiments has been heavily criticised for lacking the properties to identify the very highly (also referred to as “somnabules”) hypnotizable subjects [33]. Furthermore, the salience of special cases becomes greatly diminished when large numbers of highly susceptible persons are used and data are averaged across the group.

We suggest that the hypnotic state does not occur in all who are classified as highly-hypnotizable by using current hypnotisability scales, but only in a small subgroup of them (see also [2]). The external manifestation of those who enter the hypnotic state may be the classical Hypnotically Induced Stare (HIS). However, this result does not rule out the possibility of a hypnotic state being present without accompanying change in eye behaviour. We propose that the research field of hypnosis should also include an approach that has proven to be very effective in cognitive neuropsychology; namely using detailed case studies as one line of research to make initial theoretical progress in a relatively new and fuzzy area of empirical research [34]. This approach has previously shed new light on many rare phenomena e.g. Capgras syndrome [35] or synaesthesia [36]. Furthermore, such studies have consequently led to better general understanding of brain functioning.

However, since we only presented the results of a single case, we cannot draw conclusions about hypnosis in general or even about other hypnotic virtuosos. Further studies are needed in order to validate whether or not changes in eye movement control serve as a reliable marker of the hypnotic state in hypnosis virtuosos or in the general population. It is also possible that TS-H has somehow acquired a special ability to control her eye behavior and was in fact just using this ability during the experiment. However, we regard this as a rather remote possibility as we mostly measured automatic and involuntary eye movements (such as those occurring during the OKN), which are difficult to change through voluntary effort.

Although the present results are based on a case study, they suggest a novel way of studying and understanding hypnosis. We suggest that hypnosis is not a normally distributed psychological phenomenon in the whole population, but rather a rare and exceptional neural property or cognitive ‘skill’ found in only very few individuals. In any case, our theories and background assumptions concerning hypnosis need to be revised in the light of present results.

## Supporting Information

Movie S1 **Alteration of the state of consciousness in TS-H between baseline and hypnosis using posthypnotic suggestion.** The green text “normal state” in the upper left corner indicates that TS-H is in her normal waking state. When the experimenter (author S.K.) utters the cue “*hypno*” TS-H enters the “hypnotic state”, as indicated by the red text “hypnotic state”. As soon as the experimenter utters the cue “*base*”, TS-H returns into the “normal state” (as indicated by the green text “normal state”).
(MOV)

Movie S2 **The pattern of eye-movements of TS-H in the hypnosis condition in the Saccade task.** TS-H was asked to perform voluntary saccades from the centre of the screen (from the fixation circle) to target circles appearing abruptly in the visual periphery as fast and accurately as possible. Targets could appear at 24 different locations, at 5 different eccentricities. This video clip provides an example of TS-H's performance in this task. The eye- movements of TS-H are seen as a coloured dot. Note how the eye-movements of TS-H show a certain “creeping” like pattern.
(MOV)

Movie S3 **The pattern of eye-movements of a control subject in the hypnosis simulation condition in the Saccade task.** The participant was asked to simulate how a hypnotized person performs the task the best he/she could based on detailed descriptions (including a video clip of TS-H's eye-movements in the task) given to the subject. This video clip provides an example of one of the control subjects' performance in this task. The eye- movements of the subject are seen as a coloured dot. Note how the control subject is not able to mimic the “creeping” like pattern of TS-H's eye movements in the hypnotic state. Instead, the gaze tends to be drawn to the target directly.
(MOV)

Movie S4 **The pattern of eye-movements of TS-H in the hypnosis condition in the Partial Field Optokinetic Nystagmus (OKN) task.** TS-H was asked to maintain a stable fixation at the centre of the screen while a black-and white grating moved towards left or right at a steady pace of 4 or 6 degrees per second. This video clip provides an example of TS-H's performance in this task. The eye-movements of TS-H are seen as a coloured dot. Note how in the hypnosis condition this task elicits the optokinetic reflex to a notably smaller degree as compared to a control subject simulating the task.
(MOV)

Movie S5 **The pattern of eye-movements of a control subject in the hypnosis simulation condition in the Partial Field Optokinetic Nystagmus (OKN) task.** The participant was asked to simulate how a hypnotized person performs the task the best he/she could based on detailed descriptions (including a video clip of TS-H's eye-movements in the task) given to the subject. This video clip provides an example of one of the control subjects' performance in this task. The eye-movements of the subject are seen as a coloured dot. Note how the control subject is not able to voluntarily inhibit the optokinetic reflex (compare Movie S4).
(MOV)

Table S1 **Psychometric performance of TS-H in different tests measuring language, verbal abilities, visuospatial processing and learning/memory, executive functions and attention, and processing speed.**
(DOC)

Table S2 **The difference values (NC-HC) of TS-H and the control group (NC-HSC) in all measured variables in the Fixation task.**
(DOC)

Table S3 **The difference values (NC-HC) of TS-H and the control group (NC-HSC) in all measured variables in the Saccade task.**
(DOC)

Table S4 **The difference values (NC-HC) of TS-H and the control group (NC-HSC) in all measured variables in the Partial Field Optokinetic nystagmus (OKN) task.**
(DOC)

Text S1 **A posthypnotic suggestion.**
(DOC)

Text S2 **Neuropsychological examination of TS-H.**
(DOC)
