# Supplementary material for: The Existence of a Hypnotic State Revealed by Eye Movements
Source: PLoS One. 2011 Oct 24;6(10):e26374. doi: 10.1371/journal.pone.0026374 (PMC3200339; doi:10.1371/journal.pone.0026374)
Supplement: Table S1 — Psychometric performance of TS-H in different tests measuring language, verbal abilities, visuospatial processing and learning/memory, executive functions and attention, and processing speed. (DOC) [file pone.0026374.s006.doc]

**Supporting Information Table S1.**

**Table S1. Psychometric performance of TS-H in different tests measuring language, verbal abilities, visuospatial processing and learning/memory, executive functions and attention, and processing speed.**

| **Neuropsychological Tests** | **Score of TS-H** | **Finnish norms M** | **(s.d.)** |
| --- | --- | --- | --- |
| **LANGUAGE** |  |  |  |
| Boston Naming Test | 60 | 56.3 | (2.58) |
| WAIS-III Similarities | 25 | 21-22* |  |
| Category fluency, animals | 24 | 23.7 | (6.1) |
| **VERBAL (LEARNING AND) MEMORY)** |  |  |  |
| WMS-III Logical Memory I (immediate recall) | 52 | 43-45* |  |
| WMS-III Logical Memory II (delayed recall) | 32 | 26-28* |  |
| 30 Verbal Paired Associates (immediate recall) | 24 | 26.1 | (4.0) |
| 30 Verbal Paired associates (delayed recall) | 21 | 24.4 | (5.6) |
| **VISUOSPATIAL PROCESSING/FUNCTIONS** |  |  |  |
| WAIS-III Picture Completion | 21 | 19 |  |
| WAIS-III Block Design | 44 | 38-41* |  |
| **VISUAL LEARNING AND MEMORY** |  |  |  |
| Benton Visual Retention Test (errors) | 1 | 3.3 | (2.6) |
| Recall of 20 objects (immediate recall) | 16 | 14.3 | (2.3) |
| Recall of 20 objects (delayed recall) | 16 | 13.2 | (2.2) |
| **EXECUTIVE FUNCTIONS AND ATTENTION** |  |  |  |
| WAIS-III Digit Span (forward) | 9 | Forward +  backward 14* |  |
| WAIS-III Digit Span (backward) | 8 |  |
| Trail Making Test B (seconds) | 71 | 76.7 | (25.7) |
| Stroop colour – word interference (seconds) | 88 | 104.6 | (8.6) |
| Phonemic fluency (letter S) | 10 | 15 | (5.0) |
| WAIS-III Arithmetic | 12 | 11* |  |
| Bourdon-Wiersma Test (lines) | 34 | 33 | (5) |
| Bourdon-Wiersma Test (errors) | 3 | 13 | (5) |
| **PROCESSING SPEED** |  |  |  |
| Trail Making Test A (seconds) | 35 | 30.5 | (9.2) |
| Stroop (colour – word naming) seconds | 63 | 62.9 | (11.5) |
| WAIS-III Digit Symbol | 77 | 65-72* |  |

***In WAIS/WMS tasks the Finnish norms are often given as ranges. The ranges/scores presented here give 10 raw points referring to 50 percentile in her age group.**
