# Supplementary material for: The Existence of a Hypnotic State Revealed by Eye Movements
Source: PLoS One. 2011 Oct 24;6(10):e26374. doi: 10.1371/journal.pone.0026374 (PMC3200339; doi:10.1371/journal.pone.0026374)
Supplement: Table S2 — The difference values (NC-HC) of TS-H and the control group (NC-HSC) in all measured variables in the Fixation task. (DOC) [file pone.0026374.s007.doc]

**Supporting Information Table S2.**

**Table S2. The difference values (NC-HC) of TS-H and the control group (NC-HSC) in all measured variables in**

**the Fixation task.**

| Task performance | Fixation frequency (count/s) | Fixation duration (ms) | Blink count (count/s) |
| --- | --- | --- | --- |
| Control group Mean (s.d.) | -0,06 (0,17) | +337,3 (1077) | -0,4 |
| Best control subject performance in measured variable. | -0,45 (subject no 11) | +2877 (subject no 3) | -1,1 (subject no 9) |
| Weakest control subject performance in measured variable. | + 0,14 (subject no 8) | -1620 (subject no 1) | 0 (subject no 1) |
| The performance of the best control subject when all variables were taken into account***** (subject no 11). | -0,45 | +1935,75 | -0,7 |
| TS-H | -0,44 | + 2517 | -1,2 |
| The direction of change for control subjects between NC and HC. | 10 decreased  4 increased | 8 increased  6 decreased | 13 decreased  1 unchanged |

***The controls were rank ordered (the control subjects received points from 1 to 14) on how well they performed in**

**each variable. The subject who received most points was considered to be the best control subject in that task.**
