# Supplementary material for: The Existence of a Hypnotic State Revealed by Eye Movements
Source: PLoS One. 2011 Oct 24;6(10):e26374. doi: 10.1371/journal.pone.0026374 (PMC3200339; doi:10.1371/journal.pone.0026374)
Supplement: Table S3 — The difference values (NC-HC) of TS-H and the control group (NC-HSC) in all measured variables in the Saccade task. (DOC) [file pone.0026374.s008.doc]

**Supporting Information Table S3.**

**Table S3. The difference values (NC-HC) of TS-H and the control group (NC-HSC) in all measured variables in the Saccade task.**

| Performance in the task | Saccade amplitude (degrees) | Saccade duration (ms) | Saccade latency (ms) | Saccade velocity degrees/s |
| --- | --- | --- | --- | --- |
| Control group Mean (s.d.) | -3,2 (2,1) | -8,4 (7,6) | +159,7 (106) | -45,3 (28) |
| Best control subject performance in measured variable | -7,2 (subject no 10) | -20,6 (subject no 11) | +338 ms (subject no 3) | -102,8 (subject no 11) |
| Weakest control subject performance in measured variable | + 0,1 (subject no 8) | +1,9 (subject no 8) | -0,5 ms (subject no 8) | -8,4 (subject no 13) |
| The performance of the best control subject when all variables were taken into account* (subject no 11) | -5,83 | -20,6 | +238,3 | -70,2 |
| TS-H | -5,27 | -28 | +241 | -48,2 |
| The direction of change for control subjects between NC and HC | 13 decreased  1 increased | 11 decreased  3 increased | 13 increased  1 decreased | 14 decreased |

***The controls were rank ordered (the control subjects received points from 1 to 14) on how well they performed in**

**each variable. The subject who received most points was considered to be the best control subject in that task.**
