# Supplementary material for: The Existence of a Hypnotic State Revealed by Eye Movements
Source: PLoS One. 2011 Oct 24;6(10):e26374. doi: 10.1371/journal.pone.0026374 (PMC3200339; doi:10.1371/journal.pone.0026374)
Supplement: Table S4 — The difference values (NC-HC) of TS-H and the control group (NC-HSC) in all measured variables in the Partial Field Optokinetic nystagmus (OKN) task. (DOC) [file pone.0026374.s009.doc]

**Supporting Information Table S4.**

**Table S4. The difference values (NC-HC) of TS-H and the control group (NC-HSC) in all measured variables in the Partial Field Optokinetic nystagmus (OKN) task.**

| Performance in the task | Amount of saccades (sac/s) | Saccade amplitude (degrees) | Saccade velocity (deg/s) | Fixation duration (ms) |
| --- | --- | --- | --- | --- |
| Control group Mean (s.d.) | -0,034 (0,26) | 0.001 (0,6) | -0,25 (13,7) | -6,5 (94,6) |
| Best control subject performance in measured variable | -0,44 (subject no 14) | -1,4 (subject no 9) | -41,8 (subject no 9) | +197,8 (subject no 13) |
| Weakest control subject performance in measured variable | +0,44 (subject no 9) | +1,2 (subject no 4) | +15,9 (subject no 11) | -224,1 (subject no 1) |
| The performance of the best control subject when all variables were taken into account* (subject no 13) | -0,35 | -0,72 | -11,2 | +197,8 |
| TS-H | - 0,9 | -3,1 | -20,7 | +458,6 |
| The direction of change for control subjects between NC and HC | 9 decreased  5 increased | 6 decreased  8 increased | 7 decreased  7 increased | 9 increased  5 decreased |

***The controls were rank ordered (the control subjects received points from 1 to 14) on how well they performed in each variable. The subject who received most points was considered to be the best control subject in that task.**
