# Supplementary material for: The Existence of a Hypnotic State Revealed by Eye Movements
Source: PLoS One. 2011 Oct 24;6(10):e26374. doi: 10.1371/journal.pone.0026374 (PMC3200339; doi:10.1371/journal.pone.0026374)
Supplement: Text S1 — A posthypnotic suggestion. (DOC) [file pone.0026374.s010.doc]

**Supporting Information Text S1**

**A posthypnotic suggestion**

The definitions of hypnosis vary widely, however, typically it is a procedure where a hypnotist first gives the subject a hypnotic induction and, after this, suggestions whose nature largely depends on the nature of the situation (clinical or experimental). The hypnosis session ends when the subject/patient is “awaken” i.e. she is given suggestions about returning to her normal waking state again and feeling alert and well.

Hypnotic inductions typically have in common a withdrawal from the environmental relationships through relaxation, references to sleeping experience and concentration upon the hypnotist’s voice [1] (for examples of various hypnotic inductions, see [2]).

The suggestions given during hypnosis are typically verbally given images of states of affairs that somehow differ from the normally perceived reality. The subject’s content of consciousness is suggested to change according to the given description. The two most commonly used scales to measure the responsiveness to suggestions are the Stanford Hypnotic Susceptibility Scale, Form C (SHSS:C, [3]) and the Harvard Group Scale of Hypnotic Susceptibility, Form A (HGSHS:A, [4]). The administration of these scales follows the normal procedure of a hypnosis session. They start by general information and a hypnotic induction, which is followed by different suggestions. The procedure ends by suggestions about returning into the normal alert waking state of consciousness. The suggestions used in these scales are mostly called hypnotic suggestions, which means that they are presented after a hypnotic induction procedure i.e. *during* the assumed hypnotic state.

However, it is also possible to affect the behaviour or experience of a person *after the hypnosis session is over*. These suggestions that aim to lead to responses to be carried out after the subject is aroused from hypnosis are called posthypnotic suggestions [1,2]. These types of suggestions are often used to induce amnesia or feeling compulsion about committing a certain task after the hypnosis session. The typical formulation of a posthypnotic suggestion is a conditional statement “When A occurs then B will happen” e.g. “When you hear a tapping sound, you will reach down and touch your left ankle” [4]. These kind of suggestions have also been used in research e.g. Raz, Fan, & Posner (2005) showed a reduced Stroop-effect by giving a posthypnotic suggestion that words will look like meaningless symbols every time the participant hears the voice of the instructor [5].

The posthypnotic suggestion can also concern becoming hypnotized. The suggestion given during hypnosis is then of the type “...after this hypnosis session when you are in your normal waking state again, it is possible for you to immediately become as hypnotized as you are now. This will happen instantly after you hear me saying the cue “xxx”. This cue can be practically anything e.g. a word, a sound, a gesture etc.

The first time TS-H was hypnotized a normal standard hypnotic induction procedure by Shor and Orne (1962) was used. After she also showed immediate response to a posthypnotic suggestion about becoming hypnotized, the procedure was later used in several experiments where she has participated in [6-9].

During a hypnosis session some days before the experiment, TS-H received a standard hypnotic induction and instructions that certain words uttered by the experimenter would induce and cancel hypnosis (i.e. was given the posthypnotic suggestion). The word “*hypno*” was chosen to be the cue for entering hypnosis and the word ”*base*” for returning back into the normal state (see Movie S1 in supporting information how the “normal state” of consciousness of TS-H is altered with the “hypnotic state” by using the posthypnotic suggestion).

The subjective experience of TS-H during this procedure is that something is said to her every now and then although she does not remember afterwards what was said. She has no experience of anything special or strange happening to her during this procedure. She further describes the experience of hypnosis as feeling of calmness and relaxation. This is also a typical experience described in the literature [1]. During the hypnosis, if she is asked whether she is hypnotized or not she typically answers that she does not know or perhaps she is. TS-H can voluntarily control or decide whether she lets herself to respond to the cue about entering hypnosis.

**References**

1. Hilgard ER (1965) Hypnotic Susceptibility. New York: Harcourt, Brace & World.
2. Weitzenhoffer AM (2000) The Practice of Hypnotism. NY: Wiley.
3. Weitzenhoffer AM, Hilgard ER (1962) Stanford Hypnotic Susceptibility Scale: form C. Palo Alto, CA: Consulting Psychologists Press.
4. Shor RE, Orne EC (1962) Harvard Group Scale of Hypnotic Susceptibility: form A. Palo Alto, CA: Consulting Psychologists Press.
5. Raz A, Fan J, Posner MI (2005) Hypnotic suggestion reduces conflict in the human brain. Proc Natl Acad Sci U S A 102: 9978-9983.
6. Kallio S, Revonsuo A, Lauerma H, Hämäläinen H, Lang H (1999) The MMN amplitude increases in hypnosis: A case study. NeuroReport 10: 3579–3582.
7. Fingelkurts AnA, Fingelkurts AlA, Kallio S, Revonsuo A (2007) Cortex functional connectivity as a neurophysiological correlate of hypnosis: An EEG case study. Neuropsychologia 45: 1452-1462.
8. Fingelkurts AlA, Fingelkurts AnA, Kallio S, Revonsuo A (2007) Hypnosis induces reorganization in the composition of brain oscillations in EEG: A case study. Contemp Hypn 24: 3-18.
9. Noreika V, Falter CM, Arstila V, Wearden JH, Kallio S (in press) Perception of short time intervals in a hypnotic virtuoso. Int J Clin Exp Hypn.
