# Supplementary material for: The Existence of a Hypnotic State Revealed by Eye Movements
Source: PLoS One. 2011 Oct 24;6(10):e26374. doi: 10.1371/journal.pone.0026374 (PMC3200339; doi:10.1371/journal.pone.0026374)
Supplement: Text S2 — Neuropsychological examination of TS-H. (DOC) [file pone.0026374.s011.doc]

**Supporting Information Text S2**

**Neuropsychological examination of TS-H**

A thorough neuropsychological examination was performed on TS-H in order to examine her normal baseline neuropsychological profile and to rule out possible neuropsychological anomalies. TS-H has a normal high school and occupational background and she has not had any psychological/psychiatric disorders. She has been working as a bookkeeper throughout her working career, which is in line with her educational background. As a hobby she has studied classical music and performs actively as a member of an a cappella group of 3 women. She is also the fund manager and a member of the board of an opera association. The neuropsychological examination was performed in Finnish by a clinical neuropsychologist on the 7^th^ of April 2009.

Neuropsychological measures included in the experiment were those that are in general clinical use in Finland and together cover a wide range of cognitive functions. The tasks were administered according to standardized procedures. The neuropsychological tests included Boston Naming Test [1], Verbal Fluency Test [2], Wechsler Adult Intelligence Scale (Similarities, Digit Span, Arithmetic, Picture Completion, Digit Symbol, and Block Design subtests) [3], Wechsler Memory Scale (logical Memory I and II subtests) [4], The Benton Visual Retention Test [5], Trail-Making tests A and B [6], Stroop Test [7], Bourdon-Wiersma Test [8], 30 Paired Word Associates [9], and Recall of 20 Objects [9].

According to the neuropsychological statement, the appearance and interaction of TS-H during the interview and the testing was focused and relaxed. Her neuropsychological profile was in normal or somewhat above normal range in all assessed measures (see Table S1 for an overview of TS-H’s performance on each individual task).

**References**

1. Laine M. *et al.* (1993) Adaptation of the Boston Diagnostic Aphasia Examination and the Boston Naming Test into Finnish. Scand J Log Phon 18: 83-92.
2. Luteyn F (1966) Een nieuwe verkorte GIT. Dutch J Psychol 2: 675-682.
3. Wechsler D (1997) Wechsler Adult Intelligence Scale - Third Edition. San Antonio TX: Psychological Corp.
4. Wechsler D (1997) Wechsler Memory Scale – Third Edition. San Antonio, TX: Psychological Corp.
5. Benton AL (1963) The Revised Visual Retention Test. New York: Psychological Corp.
6. Army Individual Test Battery: Manual of Directions and Scoring. (1944) Washington DC: War Department Adjutant General’s Office .
7. Stroop JR (1935) Studies of interference in serial verbal reactions. J Exp Psychol 18: 643-662.
8. van de Loo L (1956) Enkele beschouwingen over de Bourdon-Wiersma test. Tijdschr Psychol Kring Nijmeegse Universiteit 2: 33-46.
9. Portin,R, Rinne UK (1980) Neuropsychological responses of parkinsonian patients to long-term levodopatreatment. In Rinne UK, Klinger M, Stamm G, editors. Parkinson’s Disease – Current progress, problems and management. Amsterdam: Elsevier Biomedical Press. pp. 271-304.
